# Review on anticoccidial vaccine development for chickens: Past progress, modern methods, and production bottlenecks

**DOI:** 10.1016/j.psj.2026.107875

**Published:** 2026-10-05

**Authors:** Melaku Yismaw, Getachew Terefe

**Affiliations:** a Department of Veterinary Laboratory Technology, Injibara University, Injibara, Ethiopia; b Department of Parasitology, Microbiology and Poultry Health, College of Veterinary Medicine and Agriculture, Addis Ababa University, Ethiopia

**Keywords:** Anticoccidial vaccine, Attenuation, Chickens, Recombinant DNA technology

## Abstract

Poultry coccidiosis is an economically significant intestinal disease caused by parasitic protozoa in the genus Eimeria. Developing Eimeria vaccines is a possible way to control and prevent coccidiosis. This review discusses the different methods and challenges of anticoccidia vaccine production for poultry. The production of anticoccidial vaccines involves several methods with challenges. Live Eimeria vaccines are produced using non-attenuation without any alteration or modifications in terms of pathogenicity, and attenuation methods reduce virulence through serial passage in embryonated eggs or chickens, irradiation, chemicals, heat, and live strains tolerant of ionophores. Both non-attenuation and attenuation methods are challenge, including the need for chickens’ infection to produce oocysts, high cost, and quality control tests for ensuring vaccine safety. To minimize the challenge of live vaccine production, use recombinant deoxyribonucleic acid (DNA) technology. Unlike conventional whole-cell vaccines, non-replicating subunits only contain the pathogen antigenic parts for the subunit vaccine and genetic materials for DNA vaccine production and are cloned in the presence of a harmless plasmid vector or with a harmless adjuvant. The genetic and antigenic diversity among and within species of Eimeria, chicken flocks and locations, and complex life cycle of the Eimeria species make the development of DNA and recombinant vaccines extremely difficult. Challenge infection effects in immunized chickens are determined by weight gain, feed conversion efficiency, oocyst production, and intestine lesions, but there is a lack of immunological assays for accurately predicting studies for laboratory and field trials and standard guidelines for modern poultry vaccine production in Eimeria. The production of a safe and effective vaccine against coccidiosis in poultry is crucial for minimizing economic losses and enhancing animal welfare. To select the appropriate anticoccidial vaccine development methods and assess the challenges faced, more research should be done.

## Introduction

All domestic birds, including geese, ostriches, ducks, turkeys, chickens, quails, and pheasants, are referred to as poultry. They are primarily kept for the purpose of producing meat and eggs for human consumption. The most significant of these are chickens (*Gallus gallus domesticus*) (Shakoor et al., 2021). The productivity of chickens is affected by several factors, including handling, housing, and rearing of poultry in addition to disease (Quiroz-Castañeda, 2018). However, coccidiosis caused by protozoan parasites of numerous Eimeria species (spp.), has long been recognized as a devastating disease, which has caused severe economic consequences in the chicken industry worldwide (Arczewska-Włosek and Świątkiewicz, 2014; Juárez-Estrada et al., 2023).

According to Khater et al. (2020), Eimeria spp. cause intestinal wall damage by penetrating the host's intestinal cells at several places. This leads to poor absorption, decreased weight gain, bloody diarrhea, a lower feed conversion ratio, and dehydration. Effective prevention and control measures are crucial in the fight against Eimeria infection (Cai et al., 2023). Today, the most popular preventative methods include conventional vaccinations, and prophylactic in-feed drugs (Shivaramaiah et al., 2014). However, when similar treatments were tested on different Eimeria spp., cross- and multiple-resistant drugs emerged (Khater et al., 2020) and drug residues in poultry meat and eggs (Tang et al., 2018a).

Anticoccidial vaccines have become crucial tools in the fight against coccidiosis, instead of drugs (Cai et al., 2022). Several live vaccines consisting of either virulent or attenuated Coccidian strains have been commercially developed in recent years (Blake et al., 2017). Several methods of attenuation have been used, including selection for precociousness, irradiation, chemical treatment, and serial passage in chicken embryos to decrease the pathogenicity of the parasite (Mesa-Pineda et al., 2021). Identification of protective antigens expressed during various stages of development (sporogonies, merogonies, and gametogonies) of the Eimeria parasite is essential for the development of new vaccines against coccidiosis (López-Osorio et al., 2020). Vaccines are developed by using recombinant DNA technology such as subunit vaccines, DNA vaccines and vector vaccine production (Adugna et al., 2016; Kota et al., 2017). Adjuvants are added to improve protective immunity (Lin et al., 2020).

As such, a great deal of effort has been put into developing new vaccines using modern biotechnology advancements; however, despite initial optimism, this approach has not yet resulted in a commercially available vaccine (Chapman et al., 2005), except CoxAbic® and Vac COX®, which have been marketed thus far (Gao et al., 2024). Studies have examined vaccine challenge in rodents and chickens, yet rodents' immune systems differ greatly from those of chickens (Nochi et al., 2018). In immunized chickens, a challenge infection results in higher feed conversion ratios, less oocyst shedding in feces, weight gains, less severe intestinal lesions, and higher survival rates than in the control group, but there is no standard protocol (Zaheer et al., 2022).

Commercial vaccine development has major challenges, including production costs and generating a strong immune response in chickens (Sander et al., 2019). Complex life cycles and heterologous species infections, which differ according to the different types of flocks and locations, are not simple goals to achieve (Britez et al., 2023). The development of multivalent anticoccidial vaccines for commercial poultry should be the main emphasis of future research (Chen et al., 2023). The production methods for anticoccidial vaccines are crucial for producing effective vaccines. However, there's no perfect solution, and there are advantages and challenges to all existing methods. The literature on poultry anticoccidial vaccine production challenges and methods are limited, and no studies have been conducted in Ethiopia on coccidia vaccine production methods. Therefore, the objective of this review is to review the different methods and challenges of anticoccidial vaccine production for chickens.

## Coccidia and coccidiosis

### The parasite

Poultry coccidiosis is an obligate intracellular intestinal parasitic infection, caused by the Apicomplexan protozoan belonging to the subclass coccidia, family Eimeriidae and genus Eimeria (Cunha et al., 2020). In terms of economics, the seven most significant and unique species of the genus Eimeria invade the intestinal or cecal epithelial cells. These species include*.E. acervulina, E. brunetti, E. maxima, E. mitis, E. praecox, E. necatrix,* and *E. tenella* (Shakoor et al., 2021). The parasite is host- and site- specific and differentiated based on the pathology they cause, visible macroscopic lesions, oocyst morphology, the minimum time required for sporulation, and the minimum prepatent period (Liu et al., 2023; Mares et al., 2023; Gao et al., 2024).

The life cycle of Eimeria in chickens consists of two developmental phases: an endogenous phase in the chicken intestine during which schizogony occurs followed by gametogony, fertilization, and the shedding of unsporulated oocysts, and an exogenous phase in the environment during which oocysts excreted from the chicken undergo sporulation (Khater et al., 2020) to develop into an infective stage. The thick wall of the oocyst has been associated with its bilayer structure, which is made of protein and lipid that is resistant to both mechanical and chemical damage (Fatoba and Adeleke, 2018). When exposed to moisture, oxygen, and warmth in the external environment, the oocyst will start to sporulate within about 24 h (Fig. 1) (Kim et al., 2019).

**Fig. 1:**
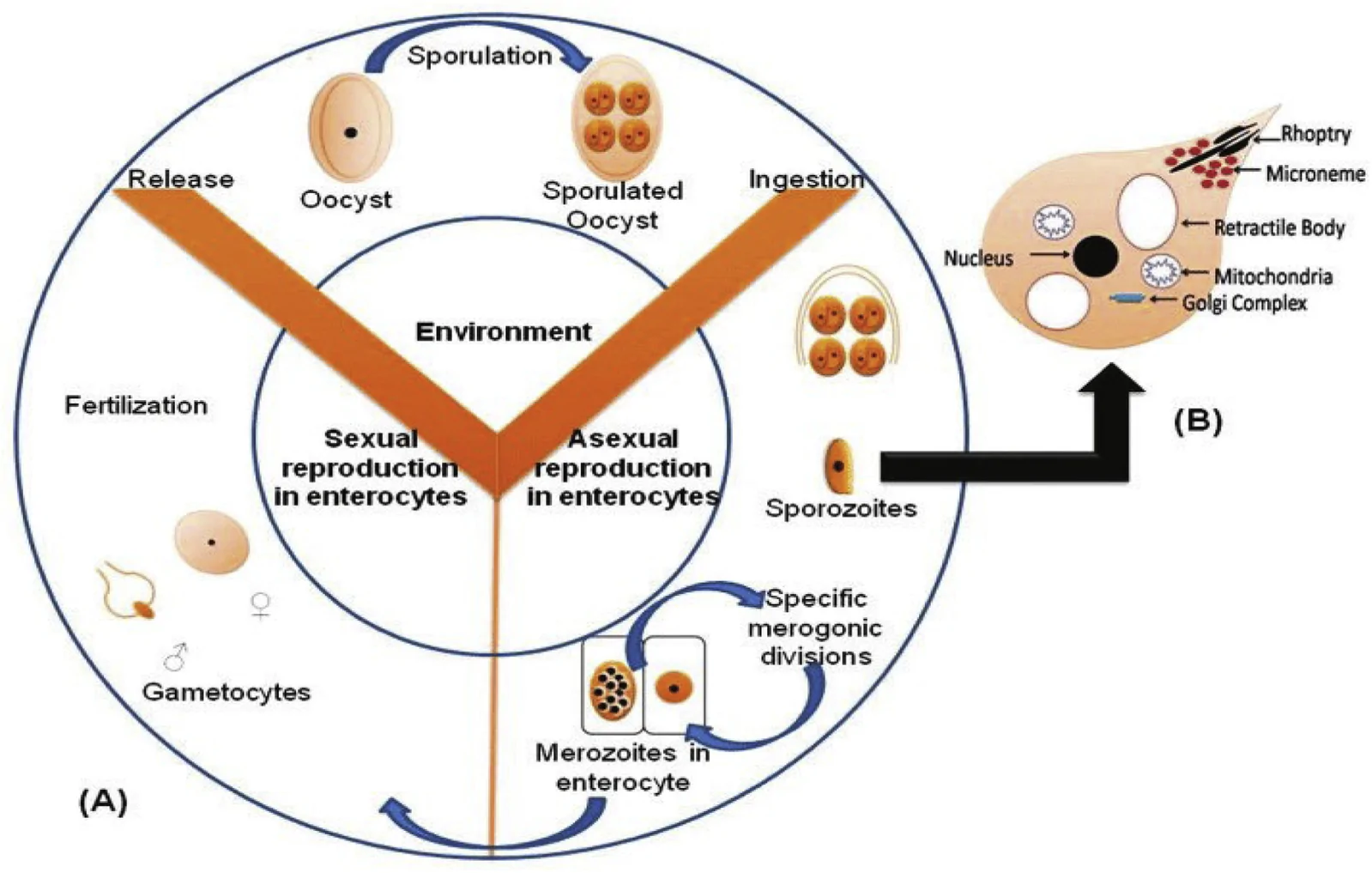
Life cycle of Eimeria. (A) Sexual and asexual stages reproduction occur in epithelium cells and oocyst formation occurs outside the chickens under specific environmental conditions. (B) Structure of Eimeria spp. sporozoite (Quiroz-Castañeda, 2018).

When the host consumes the sporulated oocyst, four sporocysts, each containing two banana-shaped sporozoites, develop within its circumplasm, signaling the beginning of the infection (López-Osorio et al., 2020). Trypsin, bile, and carbon dioxide aid in the excystation of the oocysts within the intestinal lumen, and the eight sporozoites that are liberated enter the villous epithelial cells (Yu and Heo, 2021). Sporozoites of some species (*E. brunetti* and *E. praecox*) develop within cells at the site of penetration. Sporozoites of other species (*E. acervulina, E. maxima, E. necatrix*, and *E. tenella*) are transported to other sites, viz. the crypt epithelium, where they develop as trophozoites (Tewari and Maharana, 2011). The host cell is ruptured and new ones are invaded by a high number of liberated merozoites. Male and female gametocytes can be produced from these merozoites. After fertilization, the oocysts burst open the host's enterocytes and leave with faeces (Ahmad et al., 2016; Khater et al., 2020).

### Pathogenesis and clinical features

The pathology associated with each Eimeria species varies, with infection occurring in different sections of the intestine and causing either malabsorptive (*E. acervulina, E. maxima, E. mitis* and *E. praecox*) or haemorrhagic (E*. brunetti, E. necatrix* and *E. tenella*) disease (Burrell et al., 2020). Mixed species infections are common (Andreopoulou et al., 2022) affecting different sections of chicken intestines.

Infection by a sufficiently large number of coccidia produces clinical manifestations of the disease called coccidiosis, whereas subclinical infections are asymptomatic but cause adverse effects on performance. The mildest form of infection that causes no symptoms and no adverse effects on performance infection is called coccidiasis (Mesa-Pineda et al., 2021). The sub-clinical coccidiosis manifests mainly by poor weight gain and reduced efficiency of feed conversion and gives rise to highest proportion of the total economic loss (Abebe and Gugsa, 2018).

The intestinal epithelium and underlying mucosal connective tissue are destroyed in coccidiosis, resulting in clinical signs. When symptoms become more severe, it could also accompany bleeding into the intestinal lumen (Yu and Heo, 2021). Accordingly, the clinical form of coccidiosis appears in weight loss, reduced feed intake, paleness, ruffled feathers, depression, pale color comb, wattles, huddling, closed eyes, diarrhea with bloody feces, dehydration and increased number of mortalities may accompany (Ahad et al., 2023).

### Treatment and drug resistance

Anticoccidial drugs are the most widely utilized global treatments for avian coccidiosis (Lee et al., 2022). Since 1948, chicken farming has used chemical prophylaxis to control coccidiosis (Attree et al., 2021). Anticoccidials can be classed into synthetic or chemical substances and polyether/ionophorous. As directed by the manufacturer, they were successfully applied in various combinations and concentrations for broiler and layer chickens as well as turkeys (Abd El-Ghany, 2021).

Synthetic compounds are manufactured by chemical synthesis and are often known as chemicals (Peek and Landman, 2011). Synthetic drugs work by preventing different biochemical pathways in the developing parasite's metabolism. Sulfonamides, nicarbazin, clopidol, quinolones, amprolium and halofuginone are chemical components’ examples of commonly used synthetic drugs (Chapman et al., 2010).

Ionophores/polyether antibiotics are manufactured by the fermentation of Streptomyces spp. or Actinomadura spp. and arrest the ion (i.e. sodium, potassium) transport channels and hinder the osmotic balance of the coccidian species (Peek and Landman, 2011). The groups of ionophores can be classified into three categories as follow: i) Monovalent ionophores (monensin, narasin, salinomycin), ii) Monovalent glycosidic ionophores (maduramicin, semduramycin) and iii) Divalent ionophores (lasalocid) (Yu and Heo, 2021).

Globally, widespread and extended use of anticoccidial drugs has led to the development of resistance to all of these drugs. Selinomycin, Monensin, and Amprolium resistance has previously been documented (Arabkhazaeli et al., 2013; Hunduma and Kebede, 2016; Flores et al., 2022). More precisely, because prophylactic drugs such as salinomycin and monensin are used on a regular basis in commercial feeds, there is a higher probability that resistance will develop as a result of continuous exposure (Arabkhazaeli et al., 2013; Flores et al., 2022). Different anticoccidial drugs are alternated in single and/or shuttle programs to reduce the possibility of resistance. Unfortunately, according to Peek and Landman (2011), this hasn't resolved the issue of anticoccidial resistance. Therefore, anticoccidial vaccines have become crucial tools in the fight against coccidiosis due to the growing problems of drug resistance in Eimeria and drug residues in animal-derived products (Gao et al., 2024).

## Anticoccidia vaccine

Vaccines are an effective method for preventing disease (Tang et al., 2020). B and T lymphocytes of the gut-associated lymphoid tissues may stimulate potent immune responses in the host in response to a primary avian coccidia infection (Martins et al., 2022). Chickens that have immunity to a single species of Eimeria usually have minimal or no cross-protection against other species (Gao et al., 2024).

According to the academic framework presented by researchers Ojha and Prajapati (2022) in their work, History of vaccines: from centuries to present, vaccines are divided into three distinct generational categories based on their design, manufacturing components, and safety profiles. These are: first-generation (conventional) vaccines, which consist of whole microorganisms that have been non- attenuated or live-attenuated; second-generation vaccines, also known as subunit vaccines, which are primarily made of protein components like protein antigens or recombinant proteins; and third-generation vaccines, which consist of nucleic acid (DNA and mRNA) and recombinant vector vaccines, which are made of genetic materials to elicit an immune response against a particular pathogen (Abdelaziz et al., 2024).

### Methods of Eimeria vaccine production

Vaccine development in its original form, as pioneered by Jenner and Pasteur, was involved introducing an attenuated or inactivated antigen to mimic infection and induce immunity without causing disease (Viljoen et al., 2021). All vaccines now in use are based on live virulent, and live attenuated formulations (Sharma et al., 2015), except for CoxAbic® and Vac COX®, which are subunit vaccines developed via recombinant DNA technology (Gao et al., 2024). The production of live Eimeria vaccines includes non-attenuated (wild-type strains of Eimeria spp.), attenuated (Pastor-Fernández et al., 2020) and live-tolerant to ionophores vaccines (Ahmad et al., 2016). Recombinant DNA technology is used in the production of subunit, DNA, and vector vaccines. And also used in laboratories, to create immunogenic proteins or glycoprotein subunits, which are used as recombinant vaccines (Venkatas and Adeleke, 2019).

#### Non-attenuation methods

Non-attenuated vaccines are made from wild-type strains of Eimeria spp. that have been obtained from laboratory or field strains and have not undergone any pathogenicity-altering modifications (Peek & Landman, 2011). Developed by Prof. S.A. Edgar of Auburn University and first marketed by Dorn and Mitchell, Inc. in 1952, the world's first commercially successful coccidiosis vaccine contained live, nonattenuated *E. tenella* oocysts and was sold under the trade names DM® Cecal Coccidiosis Vaccine, Coxine, NObiCOX, and CocciVac. (Zaheer et al., 2022). Oocysts, which are very effective and quite inexpensive to generate, when administered in low doses over several days, produced protective immunity against homologous challenge (Soutter et al., 2020; Mesa-Pineda et al., 2021).

#### Attenuation methods

One of the most used methods to produce chicken vaccines is attenuation (Abdelaziz et al., 2024). Since none of the species go through their whole life cycle *in vitro*, it has not been possible to reduce the virulence of chicken Eimeria spp. through serial passage in cell cultures (McDonald and Shirley, 2009). Subsequently, a number of methods for attenuating the Eimeria parasite developed, including high doses of merozoites injected intrarectally and de-routed sporozoite vaccines (Ahmad et al., 2016). As a general rule, attenuation of live parasites can be achieved through genetic manipulation (Falsafi et al., 2023), repeated passage, irradiation, chemicals, heat, and vaccines based on live strains that are relatively tolerant of ionophores (Vermeulen et al., 2001).

**Attenuation by embryo adaptation:** Eimeria spp. isolates that have undergone laboratory manipulation to reduce their virulence make up attenuated vaccines. Serial passages of the parasite in the chorioallantoic membrane of embryonating chicken eggs have been used to reduce virulence, following the inoculation of *in vitro*-released sporozoites into the allantoic cavity (Peek and Landman, 2011). When *E. tenella* is serially passed in embryonating eggs, a line that is substantially less harmful for chickens than wild-type strains emerge. This egg-adapted line of *E. tenella*, which is obtained after more than 100 passages, is utilized in the Livacox® vaccine (Liu et al., 2023).

Complete development of egg-adapted lines of *E. acervulina, E. maxima*, and *E. praecox* has not been achieved, in contrast to *E. tenella* (Shivaramaiah et al., 2014). In embryo-adapted lines of the majority of other species, McDonald and Shirley (2009) showed that *E. necatrix* do not develop because the sporozoites either did not develop after the chorioallantoic membrane invasion or led to a full endogenous cycle, but the few oocysts that were produced did not sporulate well.

**Attenuation by precocious development:** According to Tang et al. (2020), populations of Eimeria that reach their reproductive potential and endogenous life cycle in the host faster than parent strains of the wild type are known as precocious lines. Drug-sensitive, virulent strains of Eimeria spp. are frequently passed through their native host in order to select precocious lines (Shivaramaiah et al., 2014). In comparison to the parent Eimeria strain's normal life cycle, precocious strains are faster or lack one or more secondary schizogony stages (Fig. 2); they also produce fewer merozoites, shortening the pre-patent period (Table 1) and significantly lowering the number of oocysts they produce (Hu et al., 2018; Mathis et al., 2018; Chapman and Blake, 2022). Subsequently, oocysts in the feces at progressively earlier time points during oocyst shedding are collected and used as immunizing agent in chickens (Matsubayashi et al., 2016).

**Fig. 2:**
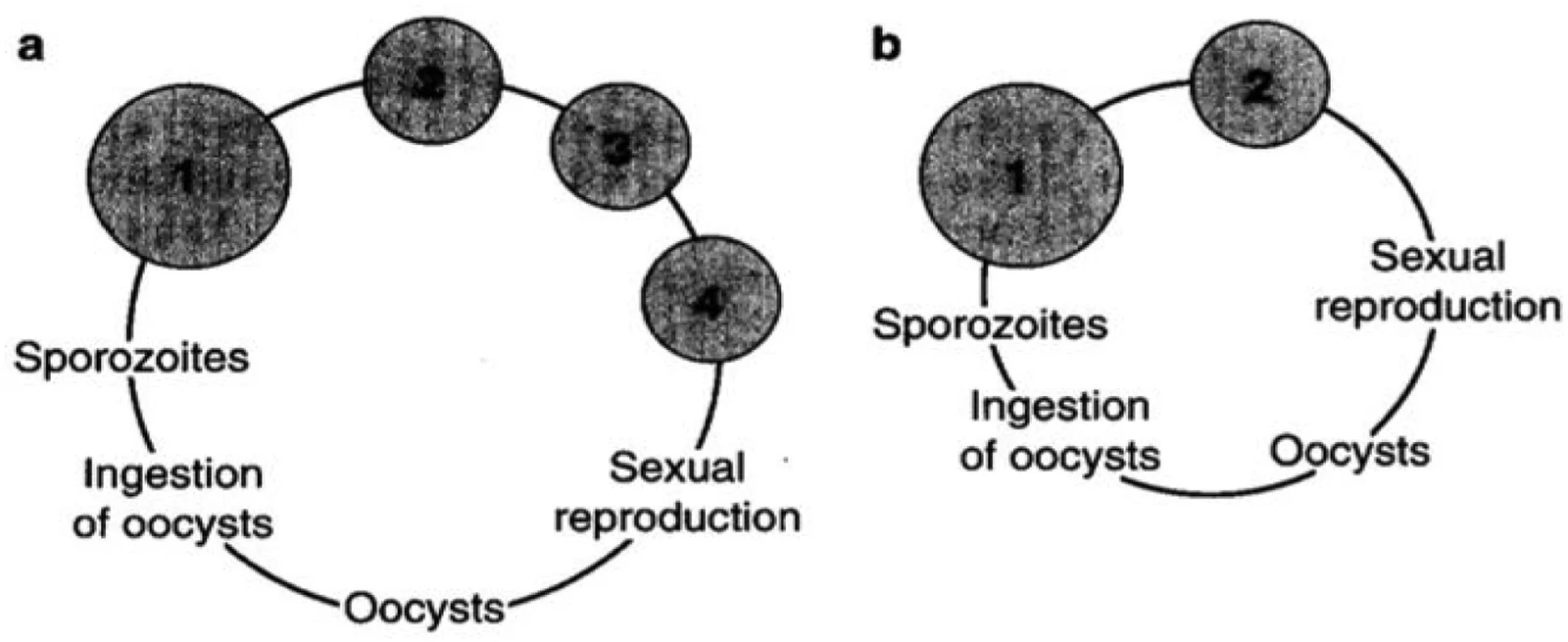
Life cycles of parent (virulent) strain of *E. maxima* (a) and a precocious line derived from it, (b) The circles numbered I-4 represent discrete generations of schizogony (asexual reproduction) occurring in epithelial cells of the intestine (Shirley and Bedrnik, 1997).

**Table 1:** Some characteristics of attenuated strains of Eimeria species from the chicken.

Species	Prepatent period (h)	Passage number	Reference	
	Attenuated	Parent		
E. acervulina	81-82	96	25	Kawazoe et al., 2005
E. tenella	72	96	20	Matsubayashi et al., 2019
*E. maxima*	98	130	22	Hu et al., 2018

Precocious parasite lines exhibit reduced pathogenicity, but importantly retain their immunogenicity (Soutter et al., 2020). Precocious attenuated strains, such as TAM™ and Neca™ (Nisseiken Co., Ltd, Tokyo, Japan) containing *E. acervulina, E. maxima, E. tenella*, and *E. necatrix*, and Paracox®-5 (MSD Animal Health, Milton Keynes, UK) which contains *E. acervulina, E. maxima, E. mitis*, and *E. tenella* are commercially available in Japan (Kawahara et al., 2014; Matsubayashi et al., 2020). On the other hand, Paracox® vaccine incorporates attenuated strains of all seven important Eimeria species affecting poultry (Chapman and Blake, 2022).

**Attenuation by chemical and irradiation:** These methods consist of parasites of artificially reduced virulence as vaccine components (Falsafi et al., 2023). However, X-irradiation and heat were unable to produce potent preparations (Ahmad et al., 2016). Diclazuril at a 20 % concentration was applied to both sporulated and unsporulated oocysts of Eimeria species for 48 h at 25-29°C. This treatment was effective in attenuating the challenge and causing a notable protective effect in layer chicks (Gadelhaq et al., 2017). A protective immunological response might be induced against local Eimeria oocysts treated with 20 % ethanol for 48 h during and after sporulation (El-Ashram et al., 2020).

Irradiation technologies, such gamma-irradiation, emerged recently as a quick and secure substitute for inactivating organisms that have little to no effect on antigenic determinants (Abdelaziz et al., 2024). Radiation attenuation preserves the pathogen's ability to multiply and maintain its metabolic activity by not destroying its protein structures (Falsafi et al., 2023). Attenuation of mixture of *E. maxima, E. acervulina*, and *E. tenella* was achieved by exposure to 150 or 200gamma radiation (Fetterer et al., 2014). Furthermore, effective attenuation by ultraviolet (250 nm) irradiation was reported by El-Ashram et al. (2019). Djemai et al. (2023) has also reported UV irradiation of *E. tenella* oocyst at 253.7nm for 1hr was effective to reduce virulence of the parasite.

*Eimeria tenella* oocytes were attenuated by radiation (100kv, 5.00mA per sec) in combination with an inulin adjuvant studied by Falsafi et al. (2023). In contrast, *E. tenella* oocysts that were subjected to brief low-energy electron irradiation between 0.1 and 10.0kilogray maintained their antigenic structures. Vaccine challenge tests show that they reduce intestine lesion scores, increase body weight gain, and improve feed conversion rate than control (Thabet et al., 2019).

#### Recombinant DNA technology

Recombinant DNA technology is crucial in vaccine development, enabling the creation of novel preventive and therapeutic vaccine candidates (Jackwood et al., 2008). It can be used to producing a subunit of pathogen antigens, latter approaches to identify the immunologically relevant vaccine component and adjuvants to enhancing immune responses (Cunningham et al., 2016).

**Conventional subunit vaccine production:** Unlike conventional whole-cell vaccines, non-replicating subunit vaccines only include the pathogen’s antigenic parts (epitopes) as immunogens (Abdelaziz et al., 2024). It utilizes safe antigens such as surface proteins or internal antigens associated with organelles such as microneme, rhoptry, and refractive proteins of sporozoites or merozoites (Venkatas and Adeleke, 2019). The first subunit anticoccidial vaccine is the CoxAbic® vaccine, a transmission-blocking vaccine. It is made up of *E. maxima* gametocyte antigens that have been affinity-purified (Gao et al., 2024). *Bacillus subtilis* was transformed using a patented Bacillus expression plasmid to produce Biotech Vac Cox (Vetanco S.A. Buenos Aires, Argentina) (Gumina et al., 2021).

**Recombinant subunit vaccine production:** Recombinant vaccines induce immune responses by utilizing specific antigens cloned from Eimeria species in the presence of a harmless plasmid vector or adjuvant (Suprihati and Yunus, 2018). Several antigens from sporozoites or merozoites have been chosen as vaccine candidates (Table 2) (Shivaramaiah et al., 2014). Nucleotide sequencing of the infectious agent's genome, identification of the gene or genes encoding the protein antigen, and insertion of the candidate gene into bacterial, yeast, or mammalian cells to express the target protein are the usual steps involved in the production of recombinant proteins. In order to produce a subunit vaccine, the produced proteins are further isolated and purified (Fig. 3) (Yin et al., 2015).

**Table 2:** Summary of chicken subunit vaccine.

Eimeria spp.	Stage	Antigen name	Vector	Adjuvant	Type of vaccine	Reference
*E. acervulina, E.maxima*	NA	Pro NetB	pcDNA3.1 (+)	Montanide IMS 106	Recombinant	Lillehoj et al., 2017
*E. maxima*	Sz	Et [EmAMA1,IMP1]	pSDEP2AIMP1S	CFA	Transgenic	Pastor-Fernández et al., 2020
	Sz	EmIMP1	pET-28a	EDA	Recombinant	Lin et al., 2020
	Sz	EmUCE	pMD19-T	CFA	Recombinant	Lakho et al., 2020
	Zoites	EmROM12,3	pET-32a(+) pVAX1	IFA	subunit DNA	Chen et al., 2021
	Sz	Et-EmPro 3-1E	pSDEP2AIMP1S	EmPro	Transgenic	Tang et al., 2018b
*E. mitis*	Sz/Mz	EmiMIC3	pET-32a	NA	Recombinant	Huang et al., 2018
*E. tenella*	NA	TA4, AGPS, AMA-1	NA	cholera toxin subunit B	Multiepitope	Fadahunsi et al., 2025
	Sz/Mz	EtCBS	pET28a	Montanide ISA 71VG	Recombinant DNA	Zhao et al., 2022
	Gam	EtGam22	pET32a (+)	Montanide ISA 71VG	Recombinant	Rafiqi et al., 2019
	Sz	EtSAG 16, 22	pET-28a pEGFP-N1	NA	Recombinant DNA	Zhao et al., 2021
	Mz Sz Sz/Mz	EtROP17 Et MIC2 EtROP27	pET30a pPIC9K pVAX-ROP27	CFA CFA NA	Recombinant Recombinant DNA Vaccine	Liu et al., 2022 Wei et al., 2025 Lv et al., 2026
*E. tenella, E. necatrix, E. acervulina* *E. maxima*	Sz/Mz	EtTA4, EnNA4, EaLDH, Em8 TA4, 3-1E, IMP1, NA4	pET28a pET32a pET28bk pPIC9K	PLGA NA	Multiepitope DNA Multivalent DNA	Song et al., 2015 Yu et al., 2022 Ma et al., 2026
*E. tenella, E. acervulina*, *E.maxima*	Sz	Ea14-3-3 EaGAPDH EaTDH EmEF-1α	pVAX1 pMD-19T pET-32a	CFA/ IFA	Multivalent DNA	Tian et al., 2017 Chen et al., 2023

Key: AGPS: Alkylglycerone-Phosphate Synthase; AMA-1: Apical membrane antigen 1; CBE: Cystathionine β-synthase; CFA: Complete freund's adjuvant; Ea: E. acervulina; EDA: Extra domain A; EF-1α: Elongation factor 1α; Ema: E. maxima; En: E. necatrix; Et: E. tenella; IFA: Incomplete freund's adjuvant; IMP: Immune mapped protein; Gam: Gametocyte; GAPDH: Glyceraldehyde-3-phosphate dehydrogenase; IMS: Immunostimulating microparticle; ISA:Incomplete seppic adjuvant; LDH: Lactate dehydrogenase; MIC: Microneme (protein); Mz: Merozoite; NA: Not available; NetB: Necrotic enteritis B-like; Np: Nano particle; PLGA: Poly (D,L-lactide-co-glycolide; Pro: Profilin; SAG: Surface antigen; Sz: Sporozoite; TDH: Transdehydrogenase; ROM: Rhomboid-like protein; ROP17: Rhoptry kinase family protein; UCE: Biquitin-conjugating enzyme; VG:Vegetable glycerides.

**Fig. 3:**
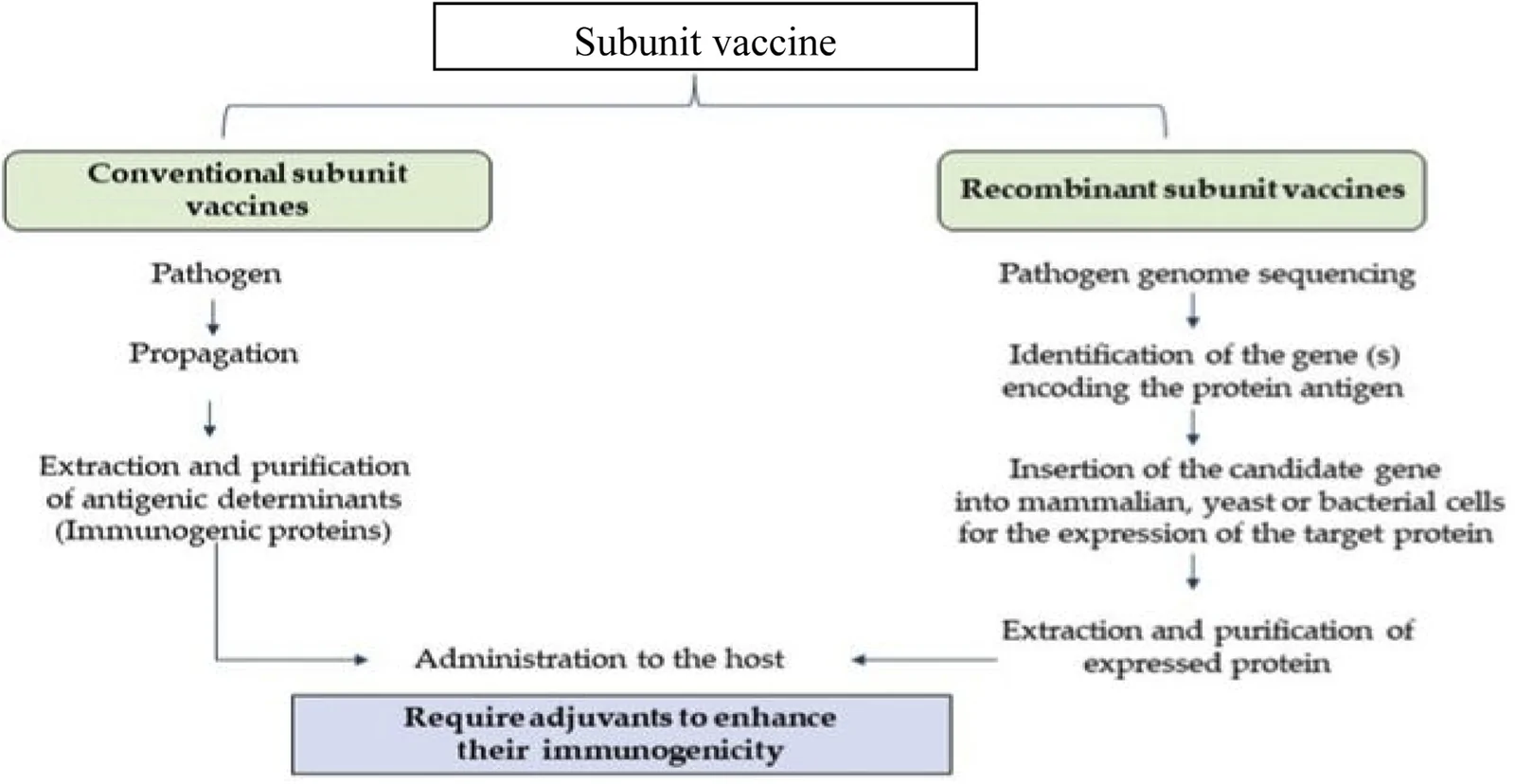
Subunit vaccine preparation (Abdelaziz et al., 2024).

**DNA vaccine production:** Third-generation vaccines use ribonucleic acid (as mRNA) and DNA (as plasmids) to allow the host cell machinery to produce target antigens, which then trigger the immune system and create memory (Abdelaziz et al., 2024). The principle is relatively straightforward: the gene encoding the immunogenic protein(s) is put into a suitable eukaryotic expression plasmid that can be purified, duplicated in bacteria, and then directly injected into the animal to be vaccinated using a variety of techniques (Fig. 4) (Shah et al., 2014).

**Fig. 4:**
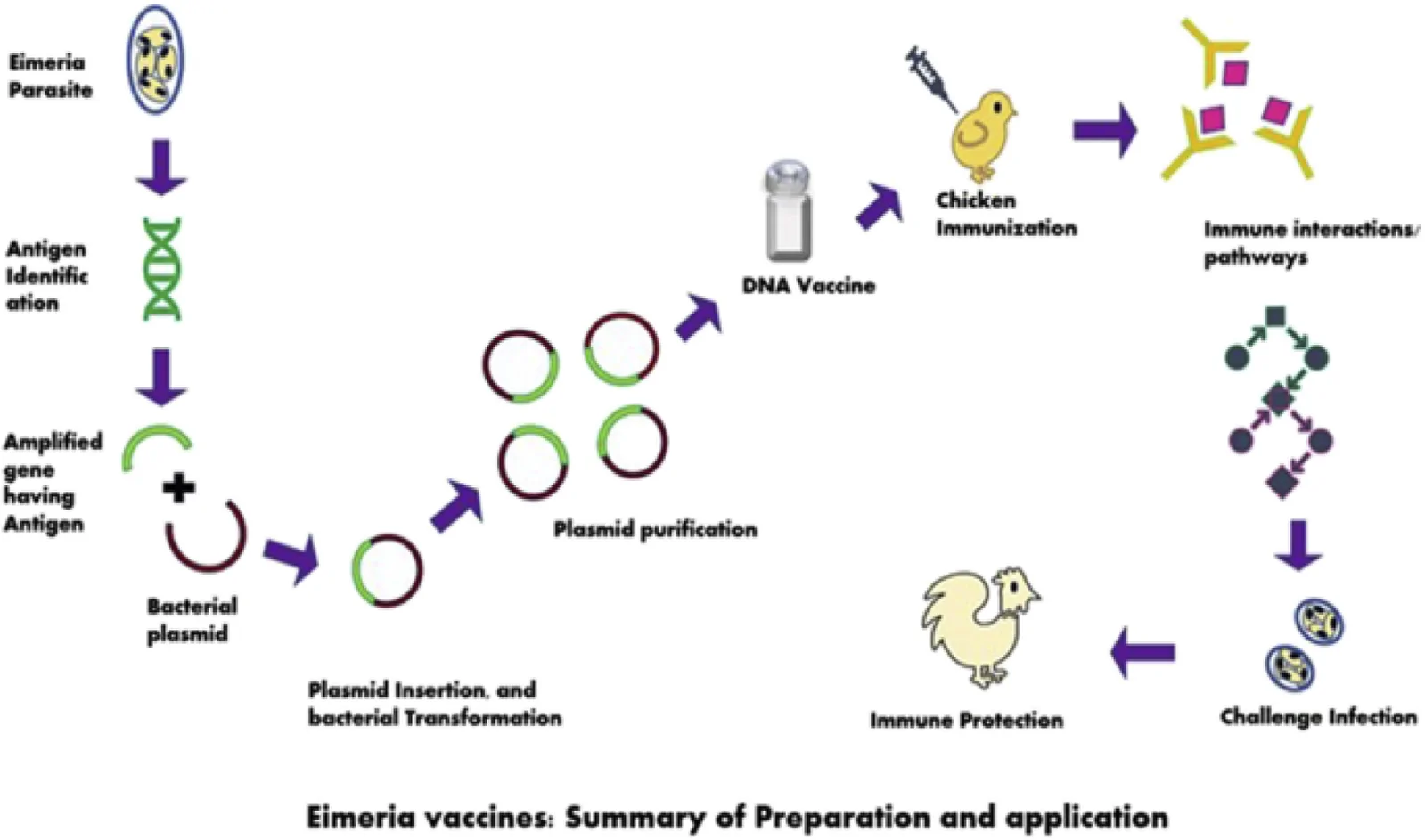
DNA vaccine preparation and application (Zaheer et al., 2022).

**Transgenic coccidian vaccine production:** Development of vector vaccine covers three steps: recombinant Eimeria selection system, exogenous antigen-specific immune response, and protection against heterologous pathogen (Duan et al., 2019). Transgenic Eimeria is constructed either through stable or transient transfection, is currently being explored as a novel delivery vehicle of Eimeria vaccine antigens (Fatoba and Adeleke, 2020). Transfected sporozoites or merozoites are transferred to the cloaca of chick, intravenously, or directly to the site where sporozoites or merozoites invade the intestine (Duan et al., 2019).

By utilizing an antigen delivery method that elicits a more extensive protective immune response and refining the antigen presentation system, recombinant and DNA vaccines can be made more effective (Trovato et al., 2015). Various types of delivery vectors including eukaryotic DNA-vaccine plasmid (pVAX1), bacterial protein-expression plasmid (pET32a) (Wang and Suo, 2020; Lee et al., 2022; Gao et al., 2024) (Table 2). Stable expression of the exogenous gene is achieved by multiple selection processes. The parasites' genomes have the ability to incorporate this foreign protein. The success rate is apparent despite several obstacles.

The development of *E. tenella* as a vector carrying the immune-dominant antigens of *E. necatrix*, reducing the need for formulations of *E. necatrix* or other pathogenic Eimeria species (Tang et al., 2019). According to Tang et al. (2018a), this delivery strategy involved the closely related species of *E. tenella* delivering the immunological mapped protein-1 of *E. maxima* (EmIMP-1) to the host immune system during the whole endogenous life cycle. Additionally, a self-destructing salmonella-based vaccine delivered through the mucosa confers protection against Eimeria, (Kong et al., 2020). However, the adenovirus- and fowl pox-vectored vaccines express *E. tenella* (Wang and Suo, 2020). Immunization with transgenic tobacco leaves expressing Gam56 and Gam82 antigens elicited a protective immune response, which was characterized by increased body weight gain and reduced fecal oocyst output (Kota et al., 2017).

### Adjuvants for Eimeria vaccines

For more than 70 years, adjuvants have been utilized to enhance the immune system's defense against antigens (Falsafi et al., 2023). The majority of adjuvants are chemicals, molecules, or particles derived from infectious pathogens or their lipid toxins, animal proteins involved in warning signals, or even compounds or proteins from plants with immunomodulatory properties (Sander et al., 2019). Several novel vaccine adjuvants have been tried for long-lasting and effective immune coverage against chicken coccidiosis (Table 2) (Lin et al., 2020). Evalon® (Hipra, Girona, Spain) is a highly effective live vaccine that uniquely benefits from the addition of an adjuvant (Falsafi et al., 2023).

Lillehoj et al. (2017) demonstrated the potential of recombinant pure profilin and NetB proteins in combination with montanide immunostimulating microparticle adjuvants to address coccidiosis. Rafiqi et al. (2018) reported similar outcomes in broiler chickens injected with rEtSO7 antigen adjuvant using montanide incomplete seppic adjuvant 71 vegetable glycerides. The *E. tenella* subunit vaccine based on yeast has been shown to inhibit Eimeria replication (Soutter et al., 2022). Zhang et al. (2012) evaluated whether combining a recombinant profilin protein with a root-extracted ginseng adjuvant (ginsenoside) could successfully prevent *E. tenella* infection. Em14-3-3 delivered by PLGA and chitosan nanoparticles conferred improved protection in chicken against *E. maxima* (Haseeb et al., 2022).

### Assessment of protective efficacy of Eimeria vaccination

The criteria used to determine the effects of a challenge infection in immunized chickens are similar to those used to evaluate drug efficacy (Chapman et al., 2005). Coccidiosis vaccine trials routinely measure host performance parameters, such as improved survival rates, body weight gains, and feed conversion ratios, alongside clinical markers like reduced fecal oocyst shedding and less severe intestinal lesions (Lee et al., 2022). Nevertheless, the field still lacks specific immunological assays to accurately predict vaccine-induced protection (Soutter et al., 2020; Nasri et al., 2022).

#### Vaccine challenge studies in laboratory trials

Animal welfare must be the priority in all research involving live animals and must be carefully planned. Replace, reduce, and refine animal utilization are the 3Rs that need to be considered (Tannenbaum and Bennett, 2015). Eimeria has inefficient *in vitro* life cycle completion capabilities. A variety of factors, such as relevance to the target chicken population, experimental design, outcomes to be monitored, test the same vaccines in different geographically distributed chicken breeds, and genetic background, are likely to influence the choice of chicken breed or line utilized in an Eimeria vaccine challenge trial (Soutter et al., 2020).

While rodent models can be used to study responses to coccidial infection, they are unlikely to completely replace the need for studies in chickens because to the fact that rodent' immune systems differ greatly from those of chickens (Nochi et al., 2018), and chicken used for challenge trial by Juárez-Estrada et al. (2021). The study design is influenced by the selection of Eimeria species. The degree of the disease and the effectiveness of immunization are affected by differences in fecundity, pathogenicity, and immunogenicity across Eimeria species. These variations also determine the size of the challenge dose and the methodology of the study (Soutter et al., 2020).

Challenge doses should be experimentally established based on the species of Eimeria and the breed of chicken. They should also be customized to the study design that the desired outcome of vaccination can be measured reliably and at an appropriate time point post infection (Nolan et al., 2015). Oral gavage of oocysts is typically used to administer the challenge dosage, which can be given as a single dose or in multiple doses to attempt to replicate a natural infection (Soutter et al., 2020). Injectable, topical, and ocular delivery methods are available for live vaccine (Chapman et al., 2005; Jenkins et al., 2013). When chicks are vaccinated with gel rather than spray, the number of oocysts shed is around seven times higher (Elnaggar et al., 2022).

According to Zhang et al. (2019), vaccinated chickens had a higher survival rate, increased average body weight gain, and a reduced gut lesion score, suggesting that DNA vaccination against *E. tenella* infection is effective. After being challenged with *E. maxima*, young chickens immunized with the Em14‐3‐3 gene from the *E. maxima* showed enhanced immunological responses, greater body weight gain, decreased oocyst shedding, and decreased gastrointestinal pathologies (Kota et al., 2017). The study's findings showed that, compared to non-immunized chickens, the 3‒1E *in ovo* vaccine produced protective immunity against *E. acervulina* infection as indicated by a lower body weight loss and a decreased fecal oocyst output (Ding et al., 2004).

#### Vaccine challenge studies on field trial

Before being approved for widespread use, all veterinary vaccinations given to animals should undergo testing for field safety and, if possible, efficacy (WOAH, 2022). No incidences of clinical coccidiosis were seen in a field trial evaluating the live attenuated vaccine Paracox®-5 for reducing broiler coccidiosis through drinking water (Claeskens et al., 2007). Similar work was done by Nowaczewski et al. (2023) on Hipracox Broilers®-Hipra. The immunized chicken showed no mortality rate compared to the control group, increased daily weight growth, a satisfactory feed conversion ratio, and a decrease in oocyst output. To determine the effect on the environment for both humans and animals, the vaccine's producers require carrying out a risk assessment (WOAH, 2022).

#### Quality control approval

A standard set of guidelines based on the modern vaccines in poultry Eimeria is lacking but some countries for avian vaccines of bacterial or viral origin can have (Chapman et al., 2005). The line of the chickens and the regimen used for vaccine in research trials may act as confounding factors increasing the statistical variability among different studies (Soutter et al., 2020).

### Challenges to produce effective anticoccidia vaccine

#### Genetic and antigenic diversity within and between Eimeria species

The development of recombinant vaccines is a challenging undertaking due to the natural genetic variability and complex life cycle of Eimeria spp. (Pastor-Fernández et al., 2018; Venkatas and Adeleke, 2019). There are two types of antigenic diversity (Smith et al., 2002). According to Ahmad et al. (2016), and, Suprihati and Yunus (2018), an infection in one subpopulation will not result in cross protection in another subpopulation that is challenged with Eimeria due to the first's involvement with antigenic diversity within the parasite. The second type is produced when large polymorphic gene families' variant proteins are switched, enabling the parasite to withstand an immune system attack in a single host (Pastor-Fernández et al., 2018). Glyceraldehyde-3-phosphate dehydrogenase from Eimeria acervulina (EaGAPDH) modulates chicken dendritic cell functions in vitro, thereby boosting Th1-type immune responses and stimulating the differentiation of autologous CD4^+^ T cells (Lakho et al., 2020).

The challenge is in the inability to quickly differentiate between field isolates from vaccinal Eimeria, as well as in diagnosing vaccine-related issues and vaccine failures (Attree et al., 2021). Genetic diversity entails the emergence of new variants among the species of Eimeria (Fatoba and Adeleke, 2020). The traditional seven chicken Eimeria species should be discussed alongside the emerging evidence for additional genetically and biologically distinct taxa such as *E. lata, E. nagambie* and *E. zaria*. Despite increasing reports of these species, their biology and epidemiology remain poorly understood. Current commercial anticoccidial vaccines do not effectively control them and cause field trial failure (Raffaelli et al., 2026). The genomic studies of Eimeria species especially *E. tenella* have indicated a wide antigenic variability that leads to the rapid development of vaccine resistance (Abd El-Ghany, 2021).

Different strains of the parasite are more common in different parts of the world (Mathis et al., 2018), and there is clear antigenic heterogeneity between the parasite's endogenous developmental stages and antigenic alteration throughout subsequent asexual generations (Sharman et al., 2010). Genetic diversity and population structure among *E. tenella* in diverse regions: India, Egypt, Libya, and Nigeria (Blake et al., 2015); genetic diversity among eight isolates of *E. tenella* from different regions of Hubei, China (Tan et al., 2017). Despite this, Kundu et al. (2017) observed that there was little nucleotide variability in the IMP1 gene of *E. tenella* in the USA, UK, China, and India.

Depending on the different types of flocks, effective vaccines against chicken coccidiosis may provide protection against several species (Britez et al., 2023). Accordingly, there is variation in the distribution of Eimeria species within and between northern and southern India based on the size, management, and system of poultry units (Prakashbabu et al., 2017).

#### Complexity of the parasite life cycle

The genus Eimeria has multiple life cycle stages. They have characteristic features such as the morphology of sporulated oocyst, the number of sporocysts per oocyst, and the number of sporozoites per sporocyst (Shakoor et al., 2021). For the development of an anticoccidial vaccine, screening for surface and secreted antigens that are strongly expressed during the sporozoite stage is thought to be important. Immunogenic proteins have been identified, isolated, and tested with some levels of success from certain stages of the life cycle of Eimeria (Shakoor et al., 2021). Different stages of Eimeria life cycle and immunogenic trials have given variable results (Gao et al., 2024). The sporozoite-specific *E. tenella* apical membrane antigen (EtAMA-1) is effective at inducing partial protection against homologous challenge with *E. tenella* when used as a recombinant protein vaccine, whereas the merozoite-specific EtAMA-2 is not (Pastor-Fernández et al., 2018).

#### Host-parasite interaction

Eimeria spp. exhibit high degrees of host and site specificity (López-Osorio et al., 2020). The multi-stage life cycle and vast genomic complexity of Eimeria create significant barriers to effective antigen discovery and vaccine development. Because the Eimeria genome encodes thousands of proteins simultaneously, profiling the parasite's complete immunogenic landscape remains highly difficult (Reid et al., 2014; Marugan-Hernandez et al., 2020). This complex, multi-protein expression profile renders host-parasite interactions unpredictable, leaving the isolation of protective antigens a persistent challenge. Historically, discovery has been hindered by bioassays that are largely restricted to extracellular stages, alongside a weak or deficient host immune response against certain critical proteins. Despite these hurdles, Yang et al. (2017) successfully identified seven genes encoding Eimeria maxima immune-protective antigens, including four novel, two recognized, and one uncharacterized gene. While various host-parasite interaction proteins have been evaluated as anticoccidial vaccines, their commercial development has stalled due to the requirements for repeated vaccinations or a failure to stimulate sufficient long-term immune protection (Pastor-Fernández et al., 2020; Feix et al., 2023).

#### Inappropriate attenuation methods for maintaining immunogenicity

The vaccine trials in chickens are challenged by lack of specific immunological assays for accurately predicting the protective efficacy of vaccines against coccidiosis (Nasri et al., 2022). The vaccines produced from live oocysts have a limited shelf life, and it might be challenging to regulate the right dosages. According to Soutter et al. (2020), there is a considerable risk of a coccidiosis outbreak from the live oocyst vaccines themselves. Developing an embryo-adapted line that achieves an optimal balance between robust immunogenicity and sufficient attenuation of virulence remains a significant challenge (Ahmad et al., 2016; Attree et al., 2021).

#### Inadequate delivery of protective antigens

An efficient adjuvant or delivery method that delivers the antigen to the appropriate place in sufficient quantities to trigger an immune response is necessary for the development of recombinant vaccines (Venkatas and Adeleke, 2019). It has been demonstrated that an oral, yeast-based subunit vaccination for *E. tenella* reduces Eimeria replication (Soutter et al., 2022). When used *in vivo*, the inherent animal adjuvants, such as chicken cytokines, are limited by their speedy host clearance and degradation (Wang and Suo, 2020).

#### Limitation of resource

There are a number of factors that restrict the production of live virulent or attenuated anticoccidial vaccines. These include the need for very large numbers of chickens to produce oocysts, quality control tests to ensure vaccine safety, time- and labor-intensive serial passaging (Zaheer et al., 2022), a short vaccine shelf life (Tewari and Maharana, 2011), the need for cold storage conditions (Blake et al., 2017; Abd El-Ghany, 2021), and limits on vaccine production capacity (Cai et al., 2022). The need for recombinant vaccines may increase as a result of such restrictions on the generation of vaccine parasite lines (Soutter et al., 2020).

The lack of cross-immunity against different Eimeria species is a significant obstacle in the production of subunit vaccines (Ahmad et al., 2016). The CoxAbic® vaccine is made by affinity purification, which is a labor-intensive, time-consuming, and costly process for removing native gametocyte antigens from parasites (Gao et al., 2024). Most of the vaccine trials in chickens typically used a single challenge dose; thus, this study design cannot address the question (Nasri et al., 2022). Limitations for developing recombinant DNA vaccines are shortage of capital, insufficiency of technology, and skilled personnel (Adugna et al., 2016). The lack of understanding of the antigens involved in powerful immunity hinders the development of recombinant vaccines against field strains (Martins et al., 2022).

#### Lack of standard protocol

A standard set of guidelines based on modern vaccines in poultry Eimeria is lacking but some countries for avian vaccines of bacterial or viral origin (Chapman et al., 2005). The lack of a standard protocol causes differences in the study designs. These variations include differences in vaccine formulation, vaccine schedule, challenge dose, and type or breed of chickens used. These variations ultimately cause the results to be heterogeneous or variable (Soutter et al., 2020). Attenuated vaccines are not as practicable because of the time and money needed for quality assurance to produce vaccine batches that meet the same requirements (Zaheer et al., 2022).

### Future perspective

To advance next-generation anticoccidial strategies, future research must shift from descriptive multi-omics profiling to the functional validation of actionable therapeutic targets. Integrated datasets spanning genomics, transcriptomics, proteomics, metabolomics, and metagenomics have successfully delineated complex host-parasite microbiome interactions within infected intestinal tissues (Reid et al., 2014). The immediate priority is to utilize these parasite stage-resolved datasets to identify highly conserved invasion and virulence factors that can serve as cross-protective antigens or tractable drug targets (Flores et al., 2026). Translating these computational models into low-cost, biosafe, and scalable vaccine formulations remains a critical prerequisite for commercial viability (Venkatas and Adeleke, 2019).

Furthermore, capitalizing on established gene-editing frameworks will require transitioning from single-gene proofs-of-concept to the development of stably attenuated, multivalent vaccine strains. While CRISPR/Cas9 systems have successfully targeted *E. tenella* ITS and 5.8S rDNA sequences to suppress oocyst sporulation and reduce lesion scores (Mohsin et al., 2021; Cheng et al., 2021), future efforts should expand this baseline feasibility (Liu et al., 2023; Qu et al., 2024) toward broader applicability. Specifically, researchers must determine whether CRISPR-mediated gene knockouts can replicate these antiparasitic effects across other economically damaging species, such as *E. maxima* and *E. acervulina*, to build a comprehensive next-generation vaccine pipeline (Gao et al., 2024).

Finally, the optimization of antigen delivery systems must focus on establishing robust, long-term mucosal immunity while addressing manufacturing and cross-species limitations. Nanoparticle-encapsulated formulations such as poly (D, L-lactide-co-glycolide) (PLGA) matrices delivering *E. maxima* lysophospholipase, regulatory T-cell inducing molecule-1, or *E. mitis* proteins have demonstrated superior immunomodulatory coverage compared to unencapsulated subunits (Xu et al., 2022; Xiang et al., 2024). Concurrently, identifying novel antigens like dense granule protein 9 (EtGRA9) has validated new avenues for reducing parasite loads (Sanchez-Arsuaga et al., 2025). The critical next step is to evaluate these recombinant proteins and Nano-vaccine delivery vehicles within large-scale, multivalent clinical trials to confirm their efficacy against heterologous field challenges (Zaheer et al., 2022).

## Conclusions and recommendations

Developing safe, cost-effective vaccines against poultry coccidiosis is critical for mitigating economic losses and improving animal welfare. Yet current non-attenuated, attenuated, and recombinant DNA platforms all present distinct operational trade-offs. While vaccine efficacy continues to be verified through standard in vivo metrics such as body weight gain, feed conversion ratios, intestinal lesion scores, and oocyst shedding. The development of Eimeria vaccines is primarily hindered by intrinsic biological barriers such as life-cycle complexity and genetic variability compounded by technical constraints in attenuation, antigen delivery, and standardized regulatory protocols. Ultimately, overcoming these bottlenecks requires the strategic integration of emerging technologies namely multi-omics profiling, CRISPR/Cas9 genome editing, and nanoparticle delivery platforms to manufacture low-cost, multivalent vaccines capable of providing comprehensive cross-species protection.

Based up on the above conclusion the following recommendations are forwarded:

✓ Cost-effective and highly reproducible vaccine production methods must be developed.
✓ Further in-depth research is needed to fully understand the biology of the Eimeria spp. for the production of vaccines.
✓ Standardized operational protocols for vaccine manufacture should be established across the industry.
✓ Future research should focus on optimizing anti-coccidial vaccine strategies and mitigating associated field and production challenges.

## Funding resource

This review article was conducted without any external funding or financial resources.

## Author contributions

All authors made substantial contributions to the conception and design of the work and/or to the acquisition, analysis, and interpretation of the relevant literature and data. All authors contributed to drafting or critically revising the manuscript for important intellectual content. All authors reviewed and explicitly approved the exact final version of the manuscript before submission. Furthermore, all authors agree to be personally and publicly accountable for all aspects of the work and to ensure that any questions concerning its accuracy or integrity are appropriately investigated and resolved.

## Disclosures

The authors declare no conflicts of interest.

## Data Availability

No new datasets were generated or analyzed.
